# Adoption of Strategies to Mitigate Transmission of COVID-19 During a Statewide Primary Election — Delaware, September 2020

**DOI:** 10.15585/mmwr.mm6943e2

**Published:** 2020-10-30

**Authors:** Eva Leidman, Noemi B. Hall, Amy E. Kirby, Amanda G. Garcia-Williams, Jose Aponte, Jonathan S. Yoder, Rick Hong, Anthony Albence, Fátima Coronado, Greta M. Massetti

**Affiliations:** ^1^CDC COVID-19 Response Team; ^2^Delaware Department of Health and Social Services; ^3^Delaware State Election Commission.

*On October 23, 2020, this report was posted online as an *MMWR *Early Release.*

Elections occurring during the coronavirus disease 2019 (COVID-19) pandemic have been affected by notable changes in the methods of voting, the number and type of polling locations, and in-person voting procedures (*1*). To mitigate transmission of COVID-19 at polling locations, jurisdictions have adopted changes to protocols and procedures, informed by CDC’s interim guidance, developed in collaboration with the Election Assistance Commission (*2*). The driving principle for this guidance is that voting practices with lower infection risk will be those which reduce the number of voters who congregate indoors in polling locations by offering a variety of methods for voting and longer voting periods. The guidance for in-person voting includes considerations for election officials, poll workers, and voters to maintain healthy environments and operations. To assess knowledge and adoption of mitigation strategies, CDC collaborated with the Delaware Department of Health and Social Services and the Delaware State Election Commission on a survey of poll workers who served during the statewide primary election on September 15, 2020. Among 522 eligible poll workers, 93% correctly answered all three survey questions about COVID-19 transmission. Respondents noted that most voters and poll workers wore masks. However, masks were not always worn correctly (i.e., covering both the nose and mouth). Responses suggest that mitigation measures recommended for both poll workers and voters were widely adopted and feasible, but also highlighted gaps in infection prevention control efforts. Strengthening of measures intended to minimize the risk of poll workers acquiring COVID-19 from ill voters, such as additional training and necessary personal protective equipment (PPE), as well as support for alternative voting options for ill voters, are needed. Adherence to mitigation measures is important not only to protect voters but also to protect poll workers, many of whom are older adults, and thus at higher risk for severe COVID-19–associated illness. Enhanced attention to reducing congregation in polling locations, correct mask use, and providing safe voting options for ill voters are critical considerations to minimize risk to voters and poll workers. Evidence from the Delaware election supports the feasibility and acceptability of implementing current CDC guidance for election officials, poll workers, and voters for mitigating COVID-19 transmission at polling locations (*2*).

Among the 2,498 poll workers who served at one of the 434 polling locations operational during Delaware’s primary election, 1,595 (64%) with valid e-mail addresses* were invited by their county elections office to complete a self-administered survey during September 23–26. Poll workers with e-mail addresses were eligible to participate if they worked on election day (September 15, 2020), were aged ≥18 years, and provided written consent to participate. Overall, 568 (36%) persons responded to the survey, among whom 522 (92%) were eligible to participate. Survey questions focused on direct observation of supply availability and polling location setup, training received, knowledge and attitudes about transmission and personal protection, and mitigation measures practiced by themselves, other poll workers, and voters. The survey was administered as a web-based Epi Info questionnaire.^†^ Data were analyzed using R statistical software (version 3.5.0; The R Foundation) and SAS (version 9.4; SAS Institute). Differences in proportions were assessed using chi-squared tests, with p-values <0.05 considered statistically significant. Industry and occupation were coded using CDC’s National Institute for Occupational Safety and Health Industry and Occupation Computerized Coding System.^§^ This activity was reviewed by the Delaware Department of Health and Social Services and CDC and was conducted consistent with applicable federal law and CDC policy.^¶^

The median age of respondents was 59 years (interquartile range = 52–69 years); 42% were aged >65 years (Table 1). The majority (57%) of respondents were male, 48% were non-Hispanic White, 42% were retired, nearly one third (32%) reported having one underlying medical condition associated with increased COVID-19 severity, and approximately one quarter (27%) reported having two or more such conditions (*3*).

**TABLE 1:** Self-reported characteristics of persons serving as poll workers during the statewide primary election — Delaware, September 15, 2020

Characteristic (no. with available information)	Respondents no. (%)
**Total**	**522**
**Gender (522)**	
Female	94 (18.0)
Male	298 (57.1)
Other/Unknown	130 (24.9)
**Age group, yrs (522)**	
Median age (interquartile range)	59 (52–69)
18–34	41 (7.9)
35–44	38 (7.3)
45–54	73 (14.0)
55–64	153 (29.3)
≥65	217 (41.6)
**Race/Ethnicity (522)**	
White, non-Hispanic	249 (47.7)
Black or African American, non-Hispanic	97 (18.6)
Other/Multiple races, non-Hispanic	9 (1.7)
Hispanic	7 (1.3)
Unknown	160 (30.7)
County of residence* (522)	
New Castle	238 (45.6)
Kent	125 (23.9)
Sussex	85 (16.3)
Unknown	74 (14.2)
**Employment status (391)**	
Retired	163 (41.7)
Employed full-time	147 (37.6)
Employed part-time	40 (10.2)
Unemployed	22 (5.6)
Self-employed	19 (4.9)
**Industry^†^ (190)**	
Public administration	49 (25.7)
Health care and social assistance	29 (15.2)
Finance and Insurance	24 (12.6)
**Occupation^†^ (184)**	
Office and administrative support	40 (21.7)
Management	21 (11.4)
Business and financial operations	21 (11.4)
**Poll worker role^§^ (481)**	
Registration desk	222 (46.2)
Greeter	127 (26.4)
Ballot processor	115 (23.9)
**Underlying medical condition (403)**	
Hypertension	129 (32.0)
Obesity	93 (23.1)
Asthma	44 (10.9)
Diabetes	36 (8.9)
One underlying medical condition^¶^	128 (31.8)
Two or more underlying medical conditions^¶^	110 (27.3)

* Delaware poll workers are eligible to serve in their county of residence.

^†^ Three most common occupations and industries coded from free text using National Institute for Occupational Safety and Health Industry and Occupation Computerized Coding System.

^§^ Poll workers often had multiple roles such that categories are not mutually exclusive.

^¶^ Underlying medical conditions assessed included asthma; autoimmune condition (such as Type I diabetes); cardiovascular disease such as heart failure or coronary artery disease; chronic liver disease; chronic kidney disease; chronic lung disease such as chronic obstructive pulmonary disease; emphysema; chronic bronchitis; cystic fibrosis; diabetes mellitus; disability (related to the brain or nervous system, intellectual, physical, vision or hearing impairment); hypertension or high blood pressure; obesity (body mass index >30 kg/m^2^); sickle cell disease; thalassemia; and weakened immune system or immunosuppressive condition (e.g., cancer, human immunodeficiency virus infection).

Physical modifications to polling locations were reported by respondents, including spacing of voting booths ≥6 feet apart (88%), modifying polling location layout such that voters moved through the space in one direction (80%), and use of visual cues to remind voters to stay ≥6 feet apart (87%) (Table 2). Use of physical barriers, such as plexiglass shields, at registration desks and between voting booths was reported by 5% and 7% of respondents, respectively. Separate doors for entry and exit were reported by 45% of respondents. In response to questions about supplies to support safe hygiene behaviors, 94% of respondents reported that hand sanitizer was available for poll workers, 82% reported that hand sanitizer was available for voters, and 93% reported that cleaning supplies were available; however, 14% reported that their polling location ran out of hand sanitizer or cleaning supplies on election day. Availability of masks for poll workers at polling stations was reported by 88% of respondents and for voters by 70%.

**TABLE 2:** Physical layout, environment, and supplies available at polling sites to mitigate COVID-19 transmission reported by poll workers during the statewide primary election — Delaware, September 15, 2020

Characteristic*	Respondents no./total no. (%)
**Total**	**522**
**No. of unique polling locations represented^†,§^**	99
**Mitigation strategies**	
**Presence of physical barriers**	
Between voter registration desk and voter check-in desks	21/462 (4.5)
Between voting booths	30/465 (6.5)
**Polling site layout**	
Layout to ensure voters move in one direction	367/457 (80.3)
Separate doors for entry and exit	204/454 (44.9)
Voting booths placed at least 6 feet apart	400/457 (87.5)
**Signs/Markings**	
Markings or decals on the floor to indicate 6 feet spacing	407/466 (87.3)
Mitigation signs in visible locations	297/465 (63.9)
**Adequate availability of supplies**	
**Polling site supplies available to poll workers**	
Hand sanitizer	394/421 (93.6)
Cleaning supplies	393/422 (93.1)
Ran out of hand sanitizer or cleaning supplies	59/418 (14.1)
Masks/Cloth face coverings	369/421 (87.6)
**Polling site supplies available to voters**	
Hand sanitizer	343/419 (81.9)
Ran out of hand sanitizer	32/413 (7.7)
Masks/Cloth face coverings	292/419 (69.7)

* Reported among persons with nonmissing response to each question.

^†^ Poll location worked not identified by 394 survey respondents.

^§^ Total of 434 polling locations were operational for the September 15, 2020 primary.

Receipt of training specific to COVID-19 mitigation was reported by 80% of respondents (Table 3). The training content most commonly reported by respondents included guidance on hand hygiene, mask use, and procedures for poll workers with symptoms. Among those respondents who received training, only 30% reported receiving training specific to assisting voters with symptoms consistent with COVID-19 or with known COVID-19 infection. Despite differences in training duration and content, 93% of respondents correctly answered all three survey questions about COVID-19 transmission,** and 94% agreed or strongly agreed that they knew how to keep themselves safe from COVID-19.

**TABLE 3:** Knowledge and practice of recommended mitigation strategies reported by poll workers during the statewide primary election — Delaware, September 15, 2020

Characteristic*	Respondents no./total no. (%)
**Total**	**522**
**Training, attitudes, and knowledge**	
**Received training specific to COVID-19 mitigation**	
Yes, received specific training	395/492 (80.3)
**Duration of training specific to COVID-19 mitigation**	
<30 mins	121/395 (30.6)
30 mins to <2 hrs	94/395 (23.8)
≥2 hrs	131/395 (33.2)
Unspecified training duration	49/395 (12.4)
**Training content**	
Procedure if poll worker suspects themselves of having COVID-19	288/376 (76.6)
Hand hygiene	281/377 (74.5)
Use of masks among poll workers	375/378 (99.2)
Assistance of sick voters	112/376 (29.8)
All content assessed^†^	65/395 (16.5)
**Knowledge and attitudes**	
Answered correctly all questions on COVID-19 transmission^§^	379/408 (92.9)
Agreed or strongly agreed that they knew how to keep themselves safe from COVID-19 as a poll worker	438/465 (94.2)
Exposures and mitigation practices among poll workers	
**Exposures**	
Reported contact with >100 voters^¶^	337/468 (72.0)
Reported close contact with >100 voters^¶^	127/465 (27.3)
Reported contact or close contact with a sick voter	19/473 (4.0)
Wore a cloth or nonmedical mask while helping sick voter	15/19 (79.0)
Wore all recommended PPE while helping sick voter**	0/19
**Mitigation practices observed among other poll workers**	
Mask use by 80%–100% of poll workers	464/470 (98.7)
Never or very rarely observed masks worn incorrectly^††^	316/433 (73.0)
Frequently or very frequently observed hand washing or use of hand sanitizer	342/437 (78.3)
Frequently or very frequently observed cleaning of high touch surfaces or equipment	395/433 (91.2)
**Mitigation practices observed among voters**	
Mask use by 80%–100% of voters	461/469 (98.3)
Never or very rarely observed masks worn incorrectly^††^	242/451 (53.6)
Frequently or very frequently observed use of hand sanitizer	193/452 (42.7)
Frequently or very frequently observed maintenance of distance from other voters	403/441 (91.4)

**Abbreviations:** COVID-19 = coronavirus disease 2019; PPE = personal protective equipment.

* Reported among persons with nonmissing response to each question.

^†^ Procedures if poll worker suspects they themselves have COVID-19, hand hygiene, mask use among poll workers and voters, use of other PPE, disinfecting high touch surfaces and equipment, maintaining physical distance, crowd management, assisting sick voters, and improving ventilation.

^§^ Knowledge score composed of three true or false questions on COVID-19 transmission that included asking whether SARS-CoV-2 can spread through respiratory droplets, when in close contact with an infected person, or from touching a contaminated surface before touching one’s face, eyes, or mouth.

^¶^ Contacts defined as within 6 feet for any amount of time. Close contacts defined as within 6 feet for a total of 15 minutes or more. The number of contacts is based on poll worker self-report.

** Recommended PPE includes respiratory protection, face shields, gowns, and gloves.

^††^ “Incorrectly” refers to not covering the mouth and nose.

Personal prevention practices were reported to have been widely adopted by poll workers and voters. Nearly all respondents (99%) reported that masks were worn by most (i.e., 80%–100%) other poll workers. A similarly high proportion of respondents (98%) reported that masks were worn by most voters. A larger percentage of respondents (73%) reported very rarely or never observing incorrect mask use (i.e., not worn over both the nose and mouth) by other poll workers compared to 54% of respondents reporting very rarely or never observing incorrect mask use by voters. In addition, a larger percentage of respondents reported frequently or very frequently observing hand sanitizer use among poll workers (78%) than reported observing hand sanitizer use among voters (43%). As well, 91% of respondents reported frequently or very frequently having observed fellow poll workers cleaning high touch surfaces and equipment. Nearly all (91%) respondents reported frequently or very frequently observing voters maintaining ≥6 feet of distance from one another.

Nearly three quarters (72%) of respondents reported contact (within 6 feet) with >100 persons and 27% reported close contact (within 6 feet for ≥15 minutes) with >100 persons on election day. Only 19 (4%) of 522 respondents reported knowingly having had contact with a person identified as being ill (with or without a known COVID-19 diagnosis); 15 of those persons reported having worn a mask during contact with the ill voter, but none reported wearing all PPE (respiratory protection, face shields, gowns, and gloves) recommended in interim guidance (*2*).

As a proxy for total voters per polling location, experiences of respondents reporting contact with >100 persons were compared with those of respondents reporting fewer contacts for all analyses of mitigation strategies, training, knowledge and attitudes, and exposures. Among respondents indicating polling location worked (128), at least 99 unique sites (23% of all operational polling locations) were represented. Availability of separate doors for voter entry and exit was reported by 37% of respondents having contact with ≤100 persons, compared with 48% of those having >100 contacts (p = 0.02). Compared with respondents having contact with >100 persons, those having contact with ≤100 persons were more likely to report very rarely or never observing voters wearing masks incorrectly (63% versus 49%, p = 0.01). No other statistically significant differences were observed.

## Discussion

The Delaware Department of Elections reported that 177,529 persons cast ballots during the 2020 primary election, nearly twice the number who voted during the 2016 primary (94,039) (*4*). Among all persons who cast ballots, 101,135 (57%) voters cast ballots in person on election day in 2020, compared with 89,280 (95%) voters in 2016 (*4*). Poll workers serving during the 2020 Delaware primary election included a large proportion of persons at increased risk for severe COVID-19–associated illness, with 42% aged >65 years and 59% having at least one underlying medical condition. The age distribution observed among survey respondents was similar to that of poll workers in Delaware during the 2016 general election, when 45% were aged >61 years (*5*). Population-based surveillance data suggest similar prevalences of underlying medical conditions among survey respondents and adults in the general population for most common conditions (*6*). Ongoing efforts to recruit younger poll workers might reduce the proportion of poll workers at risk for severe COVID-19–associated illness.

Reported infrastructure and mitigation practices generally aligned with CDC guidance for mitigating transmission of SARS-CoV-2, the virus that causes COVID-19. Most respondents reported availability of masks for poll workers as well as recommended supplies for hand hygiene and disinfection. Supplying masks for voters, although not explicitly recommended in interim guidance, might support adoption of personal prevention practices among voters. Similarity in observations related to most mitigation measures by respondents who had contact with a large number of persons and those who had contact with fewer persons at their polling locations suggests that these findings might be applicable in both smaller and larger polling locations.

This analysis identified areas where infection prevention measures could be improved in upcoming elections. The large number of close contacts (≤6 feet for ≥15 minutes) reported by poll workers underscores the potential for in-person voting locations to serve as mass gathering events, supporting current guidance related to the importance of absentee voting, extended polling location hours, and other voting options that reduce congregation of voters in polling locations. With respect to in-person voting, adoption of physical barriers and separate entrances and exits can support physical distancing; however, limited options in terms of polling locations and other physical or regulatory challenges might affect the ability to adopt some of these measures. Interim guidance recommends alternative voting options (e.g., curbside voting) and use of PPE when assisting a voter with symptoms or known infection (*2*); however, survey responses suggest that poll workers did not use recommended PPE in this setting and had limited training concerning its use*.* In settings with community spread, infection control measures should be followed, presuming that ill voters might have COVID-19. Ensuring that ill voters can vote while maintaining poll worker and voter safety will be essential to minimizing transmission without restricting voting rights. In April, Delaware began mandating mask use among persons aged >12 years, and in a July survey, approximately 79% of persons in all Delaware counties reported always wearing a mask in public when in close contact with other persons (*7*,*8*). Results from this survey indicate that the majority of both voters and poll workers wore masks at polling locations during the September primary. However, the substantial proportion of respondents who reported observing incorrect mask use by voters (i.e., masks not covering the nose and mouth) suggests that further messaging on proper mask use, including at polling locations, might be needed to strengthen the effectiveness of masks during upcoming elections.

The findings in this report are subject to at least four limitations. First, the final sample included 21% of all poll workers serving during the primary. Exclusion of persons without valid e-mail addresses and nonresponse among eligible poll workers might have biased the sample. Second, results from the Delaware primary might not be generalizable to other states or future elections; adoption of mitigation strategies could be affected by differences in COVID-19 incidence, knowledge of COVID-19 transmission, voter turnout, and differences in voting practices by jurisdiction. Third, findings assessed only mitigation practices during in-person voting on election day, although findings would also be relevant to in-person early voting. Finally, these findings are based on poll worker reports rather than direct observation and might be subject to recall and social desirability biases.

Adherence to mitigation measures is important not only to protect voters but also to protect poll workers, many of whom are older adults. Evidence from the Delaware election supports the feasibility and acceptability of implementing current CDC guidance for election officials, poll workers, and voters for mitigating COVID-19 transmission at polling locations.

**Summary**

**What is already known about this topic?**

CDC has published interim guidance for elections officials, poll workers, and voters for maintaining healthy environments and operations at polling stations to mitigate SARS-CoV-2 transmission.

**What is added by this report?**

Survey responses from Delaware’s September 15, 2020 primary election poll workers demonstrate the feasibility of implementing CDC guidance, but highlight the large number of persons poll workers have close contact with as well as gaps in infection prevention, including ensuring correct mask use and providing training and personal protective equipment to poll workers assisting ill voters.

**What are the implications for public health practice?**

Enhanced attention to reducing congregation in polling locations, correct mask use, and enabling safe voting options for ill voters are critical considerations for elections to minimize risk for voters and poll workers.

## Footnotes

* E-mail addresses were considered valid if nonmissing, without obvious typographic errors, and did not return an automated error message.

† https://www.cdc.gov/epiinfo/index.html.

§ https://wwwn.cdc.gov/nioccs3.

¶ 45 C.F.R. part 46, 21 C.F.R. part 56; 42 U.S.C. Sect. 241(d); 5 U.S.C. Sect. 552a; 44 U.S.C. Sect. 3501 et seq.

** Respondents were asked three true or false questions regarding whether 1) SARS-CoV-2 can spread through respiratory droplets, 2) SARS-CoV-2 can spread when in close contact with an infected person, and 3) SARS-CoV-2 can spread by touching a contaminated surface before touching one’s face, eyes, or mouth.
